# “When the safe place is not safe”: therapists’ perspectives on implementing TF-CBT during the war in Ukraine

**DOI:** 10.1186/s13034-026-01142-0

**Published:** 2026-08-16

**Authors:** Maike Garbade, Mariia Hrynova, Cedric Sachser, Elisa Pfeiffer

**Affiliations:** 1 Department of Clinical Psychology and Child and Adolescent Psychotherapy, Catholic University Eichstätt-Ingolstadt, Ingolstadt, Germany; 2 Clinic for Child and Adolescent Psychiatry, Psychosomatics and Psychotherapy, Ulm University, Ulm, Germany; 3 German Center for Mental Health (DZPG), Partner Site Ulm, Ulm, Germany; 4 Department of Clinical Child and Adolescent Psychology, Institute of Psychology, University of Bamberg, Bamberg, Germany

**Keywords:** Trauma, Children, PTSD, Ukraine, War, TF-CBT, Ongoing war, Trauma-focused treatment

## Abstract

**Background:**

Since the beginning of the Russian war against Ukraine in 2022, various evidence-based trauma-focused therapies have been implemented in a region that was previously structurally underserved. Despite the high demand for psychotherapeutic care, little is known about the practical implementation of these therapies under conditions of war. The aim of this qualitative study was to explore the experiences of Ukrainian therapists in delivering Trauma-focused Cognitive Behavioral Therapy (TF-CBT) for children and adolescents during the war and to identify benefits, specific challenges and necessary adaptations.

**Method:**

In April 2023, three focus groups were conducted with a total of seven Ukrainian therapists who were part of the “TF-CBT Ukraine” project. The focus groups were transcribed, translated into English, and analyzed using qualitative content analysis with MAXQDA.

**Results:**

All participants had already initiated or successfully completed TF-CBT treatments and reported positive therapeutic experiences underscoring the acceptability and perceived benefit of the intervention under highly adverse conditions. At the same time, a range of challenges was identified, including an unstable security situation, unreliable internet connections, frequent relocation of clients, and difficulties in working with parents. Therapists discuss possible reasons for this, like the parents’ own psychological burden or the limited mental health literacy within the general population. Therapists described specific adaptations in their therapeutic practice to address these challenges, including adjustments to pacing, and enhanced efforts in psychoeducation and stabilization.

**Conclusion:**

The findings indicate therapists perceive TF-CBT as generally feasible under conditions of war, highlighting its robustness as an evidence-based intervention in humanitarian settings. However, the results also emphasize the necessity of context-specific adaptations to address ongoing instability, structural barriers, and sociocultural factors. Beyond the Ukrainian context, these findings provide valuable implications for the implementation and scaling of trauma-focused interventions in other war- and crisis-affected regions. They further underscore the importance of sustained training, supervision, and support for therapists to ensure treatment fidelity, as well as the need to integrate mental health literacy initiatives at the community level to maximize both effectiveness and long-term sustainability.

**Supplementary Information:**

The online version contains supplementary material available at 10.1186/s13034-026-01142-0.

## Introduction

Wars and conflicts pose a high risk to the mental health of affected populations worldwide [5]. The ongoing large-scale Russian invasion of Ukraine has deprived millions of children and adolescents not only of their safety but also of their psychological stability. The mental health burden among Ukrainian children and adolescents has already been documented in several studies with rates of post-traumatic stress symptoms (PTSS) between 12.6% to 70% [13, 22, 26] and 32% screened positive for moderate to severe depression, and 17.9% for moderate to severe anxiety [13]. Therefore, providing effective mental health care support is of central importance in order to reduce the mental health burden of traumatized children and adolescents in Ukraine.

Until March 2022, no trauma-focused evidence-based treatments for children and adolescents had been systematically implemented and evaluated in Ukraine [14, 16]. Therapists lacked of knowledge about existing treatments [16]. An internationally recognized and evidence-based method for treating post-traumatic stress disorder (PTSD) in children and adolescents is “Trauma-Focused Cognitive Behavioral Therapy” (TF-CBT) [6]. TF-CBT has already been successfully applied and scientifically evaluated in many contexts and countries across the globe, including low- and middle-income countries or conflict-affected populations [32, 33]. Particularly in work with conflict-affected populations, meta-analyses identified TF-CBT as the most effective treatment for complex trauma, demonstrating superior outcomes compared to other therapeutic approaches or larger evidence base [20]. Cohen et al. [7] have proposed several strategies how to apply TF-CBT for youth who experience ongoing trauma, nevertheless, there is currently very limited empirical knowledge about whether and how TF-CBT can be applied under the conditions of an ongoing war. Therefore, the online-based training program *“TF-CBT Ukraine”* was developed and has been implemented in Ukraine since 2022 [24]. The aim of the program was to train Ukrainian therapists in TF-CBT and thereby make TF-CBT available to potentially traumatized children and adolescents in Ukraine. In total, *N* = 138 therapists participated in the program and *n* = 62 were certified as TF-CBT therapists [25]. The systematic evaluation of the training program demonstrated high participation rates, positive satisfaction ratings and positive outcomes at both the therapist and patient levels [25]. So far, the implementation of TF-CBT in Ukraine has primarily been documented through quantitative data and qualitative findings remain limited. Nevertheless, the perspective of therapists is particularly important, as it can provide essential insights for the planning of future implementation programs, not only in Ukraine but also in other war and crisis regions worldwide. Qualitative investigations can contribute to a better understanding of how the war context affects both training and treatment processes. Beyond methodological considerations, the implementation of evidence-based interventions in conflict-affected settings is also shaped by broader cultural and societal factors. These include mental health literacy, stigma, and attitudes toward psychological treatment, which may influence both acceptance and uptake of psychological interventions [1]. In the Ukrainian context, recent studies have documented persistent mental health stigma as well as changing help-seeking behaviors during the ongoing war [11, 21]. In addition, caregiver perceptions of child mental health and parenting practices in the context of armed conflict may further shape engagement with trauma-focused interventions [2, 9].

Hence, this study aims at examining the following research questions via a qualitative study design (focus groups):

1. Which specific challenges in implementing TF-CBT do Ukrainian mental health care professionals encounter during the ongoing war?
2. How do the therapists describe adapting TF-CBT to the specific contextual demands of the war setting?
3. How do the therapists perceive the training program in supporting the implementation of TF-CBT under these circumstances?

This study aims not only to make an important contribution to the understanding and further development of trauma-focused therapy measures in crisis regions, especially in Ukraine, but also to specifically give a voice to those who provide psychotherapeutic care and support to traumatized children and adolescents under war conditions.

## Methods

This study is part of the project 'TF-CBT Ukraine' [24], in which 138 Ukrainian therapists participated in a large training program on TF-CBT between 2022 and 2024 [18, 24–26]. The project received ethical approval by Ulm University in Germany (Number: C1/Sta) and the Zhytomyr Ivan Franko State University (Number: 9–08072022) in Ukraine.

### Trauma-Focused cognitive behavioral therapy

The evidence-based trauma-focused treatment TF-CBT consists of nine components summarized by the acronym PRACTICE [6]. These include (1) psychoeducation and parenting skills (P), relaxation (R), affective modulation (A), cognitive coping (C), trauma narrative (T), in vivo exposure (I), conjoint parent–child sessions (C) and enhancing safety and development (E). The authors of the treatment approach recommend a minimum of eight sessions to ensure that all aspects of the manual are adequately addressed. The involvement of caregivers constitutes an essential component of TF-CBT, according to Cohen et al. [6], caregivers should participate in all sessions. Each session lasts 90 min, with 45 min allocated respectively to the child/adolescent and the caregiver.

### Training-Program “TF-CBT Ukraine”

Within the framework of the training program “TF-CBT Ukraine,” 138 Ukrainian therapists were trained in TF-CBT between 2022—2026. Of whom 97.1% were female, had a mean age of 39.6 years (SD = 8.8) and an average of 7.6 years of clinical experience. Most participants were psychologists (88.4%), 63.8% reported a CBT background, and 86.2% were located in Ukraine at training onset. The online-based training program “TF-CBT Ukraine” comprised the following mandatory components: (1) self-study of the TF-CBT manual and/or completion of the web-based training; (2) participation in a three-day online training; (3) participation in at least 10 monthly supervision sessions over the course of one year; and (4) provision of TF-CBT treatment to at least three children or adolescents from Ukraine. Upon successful completion of all training components, participants received their official TF-CBT therapist certification. Of the therapists who attended the basic training, approximately half completed all training requirements and obtained TF-CBT certification. For a more detailed description of the sample, please see Pfeiffer et al. [25].

In addition, optional workshops were offered on the following topics: traumatic grief, trauma assessment, related measurements in the field of trauma treatment, caregiver involvement, strategies for implementing TF-CBT during ongoing trauma exposure, sexual development and sexually problematic behavior in children and adolescents, treatment of depression in the context of trauma, and suicidality. Furthermore, all therapists had the opportunity to participate in the PRACTICE Skills Course aimed at enhancing their personal and professional well-being [27].

All program components were delivered by certified international TF-CBT trainers or, in the case of the optional workshops, by international experts. Since August 2022, all training content has been simultaneously translated into Ukrainian. In addition, a wide range of therapeutic materials were translated and made available to participants. For a more detailed description of the Training program, please see Pfeiffer et al. [24].

### Recruitment and participants

In March 2023, a total of *N* = 28 Ukrainian therapists who were enrolled in the project “TF-CBT Ukraine” were invited via email by the study team to participate in a focus group to share their experiences in implementing TF-CBT with Ukrainian children and adolescents. This invitation was sent exclusively to therapists who had previously provided TF-CBT treatment to children during the project and were recommended by the respective trainers and supervisors were asked to identify potential participants who had demonstrated consistent engagement and active involvement in the project's activities. Of those invited, *n* = 13 (46%) therapists agreed to participate. All participants provided informed consent for study participation, video recording, and the anonymized analysis of the focus group data. Of the therapists who registered, a total of *n* = 7 (100% female) ultimately participated in the focus groups. Of those, six were Psychologists and one Psychiatrist. At the start of the training program, they were located in 7 different cities in Ukraine (Kyiv, Dnipro, Mokolaiv, Zaporizhzhia, Lutsk, Lwiw and Schytomyr).

### Data collection

In alignment with the research questions, a semi-structured interview guide comprising open-ended questions was developed (see ESM1). The formulation of the questions was informed by findings from the above presented previous research findings and the authors’ expertise in the project management of the "TF-CBT Ukraine" project [24]. In this context, not only were the authors’ experiences gained through direct project work with the participants taken into account, but also the written and oral feedback from participants of “TF-CBT Ukraine,” as well as the input provided by the international trainers. To ensure cultural appropriateness, the interview guide was reviewed by MH, a Ukrainian psychologist residing in Germany and a member of the research team.

While four focus groups were initially planned, only three were conducted due to organizational constraints. However, prior research suggests that two to three focus groups are typically sufficient to identify approximately 80% of relevant themes [15]. Therefore, the number of focus groups conducted is considered adequate for addressing the present research questions. All three focus groups took place in April 2023. In Focus group 1, only one therapist participated, in Focus group 2 and 3 there were 3 therapists participating in each group. Each focus group was conducted online and facilitated in Ukrainian by MH, an Ukrainian female psychologist and researcher, who was also in training of TF-CBT. All sessions were video recorded. Each focus group lasted up to 130 min (*M* = 111 min, *SD* = 30,92; Range 75 – 130 min). Only the moderator and the participants were present during the focus groups.

At the beginning of each session, the moderator and participants introduced themselves. The moderator then provided an overview of the focus group structure before initiating the discussion with the first question. Throughout the session, the moderator actively encouraged participation from all group members to ensure a balanced and inclusive discussion.

### Data analysis

The data were transcribed and anonymized by MH, a Ukrainian native speaker and translated into English by professional Ukrainian interpreters who are familiar with TF-CBT. The data was analyzed according to Kuckartz and Rädiker [19] by two authors (MG and a research assistant (VP)), one of whom (MG) holds a master degree in intercultural psychology and migration studies and the research assistant is currently finishing her master studies in clinical psychology. After an initial familiarization with the content of the transcripts, the coding process combined inductive and deductive approaches, supplemented by analytical memos to discuss unclear expressions. Based on the interview guideline, MG developed a preliminary codebook from all three focus groups, which was refined through ongoing analysis with input from VP, reaching consensus on code after review. Discrepancies in coding were discussed and resolved through consensus within the research team. In cases of divergent interpretations, the respective passages were re-examined in their original context until agreement was reached. Negative or deviant cases were systematically identified and analyzed in order to refine the coding framework and to ensure that the final code list adequately captured variations and contradictions within the data. The resulting defined code list was analyzed using MAXQDA 24. Codes that were mentioned only once were grouped under the code “other”.

## Results

**Fig. 1:**
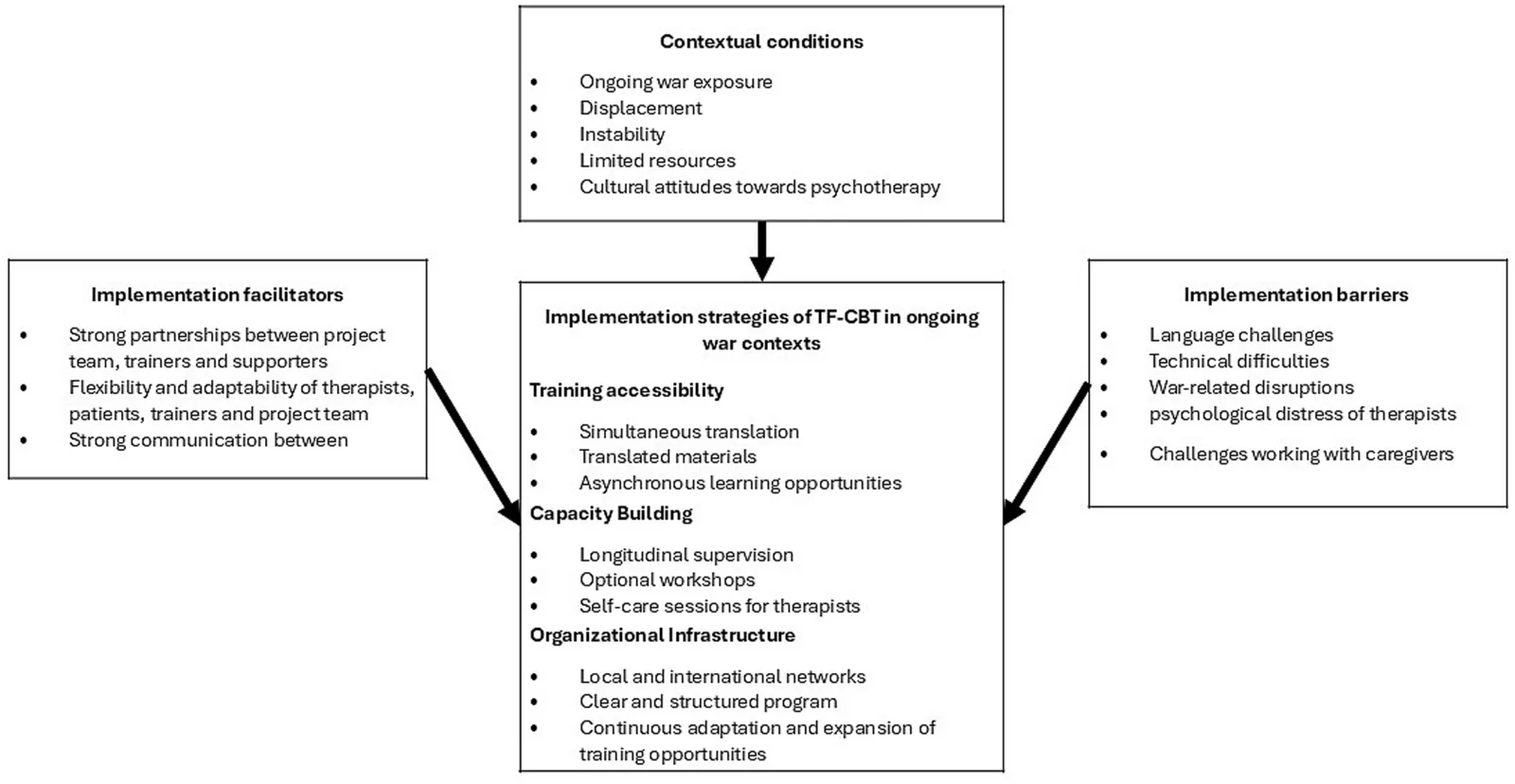
Conceptual framework

Integrating the themes and subthemes derived from participants’ accounts, we developed a conceptual framework illustrating how TF-CBT implementation unfolds within the context of an ongoing war. The framework incorporates implementation strategies, contextual factors, and key facilitators and barriers identified in the qualitative analysis. Figure 1 presents the framework as an integrative representation of the relationships among the identified domains and the qualitative findings.

## Experience in implementing TF-CBT in Ukraine

None of the therapists had to actively recruit patients themselves. Instead, existing networks were utilized, such as contacts with local social services, other therapists, or institutions like Non-Governmental Organizations, juvenile criminal police, or churches, which referred patients to the therapists. Nevertheless, the participating therapists mentioned that there is a substantial unmet need for therapy among children and adolescents from Ukraine, many of whom have not yet received treatment.

Some therapists previously had little experience in conducting psychotherapy with children and adolescents, as they had primarily worked with adult clients. Since working with children requires a different approach than with adults, various adjustments were made, such as allowing children to bring toys or pets to therapy sessions, incorporating movement breaks during sessions, or using creative methods like drawing instead of relying solely on verbal narration of experiences. ID 1.1. stated: *“During our sessions, I also allow children to get up and move. I say, ‘It’s been fifteen minutes now. Let's jump around.’ They love it. Body activation is a must for children. And then, “Can I show you a break dance?” – “Okay”. Why not give a child two minutes? (Position 1.117)“.*

All therapists reported subjective improvements in patients' symptoms, which became particularly evident through feedback from parents to therapists. Improvements in sleep, renewed participation in daily activities such as attendance at school or participation in hobbies, shared meals with the family, and increased social contact were frequently mentioned indicators of symptom improvement:

*“Back to this traumatized boy, we are already reaching the end of our therapy, and his mother wrote a very nice message to me last week saying that her child had been suffering for a year, and now he could finally sleep. This is the first month that he has been sleeping through the night. Before that he had disturbing dreams and couldn't fall asleep.” (ID 2.1, Position 2.163).*

However, therapist ID 1.1 also reported that there were some of her patients whose symptoms became more severe following exposure than they had been before (Position 1.252).

TF-CBT was conducted both in-person (7/7 therapists) and online (6/7 therapists), depending on the professional circumstances of the therapists and the geographical locations of the patients. Therapists clearly expressed a preference for in-person sessions, as they allowed for a more accurate response to patients' physical reactions and were not affected by poor internet connectivity, which could otherwise disrupt the flow of a therapy session. Nevertheless, therapists also acknowledged the benefits of online therapy, as it enabled access to patients located in other countries or regions remote areas. As one therapist put it, “*it's better to do it [online] than not to do it* (ID 3.2, Pos. 3.196)”. Furthermore, the children were according to the therapists already familiar with the online format due to the COVID-19 pandemic, which facilitated the online TF-CBT implementation. From the therapists’ perspective, however, ID 1.1 clearly articulated a desire to learn more about how specific situations, such as working with parents or with sexually abused children, can be implemented effectively and safely in an online setting, since she was lacking knowledge in this regard (Position 1.92).

Having a clear structure for the therapy sessions through the manualized intervention was perceived as very helpful. ID 2.1 describes it as follows:

*“The first thing that I really like and use with my patients is that there is a therapy plan that I follow, there are clearly structured sessions. I know exactly what I am doing. I can explain everything to my clients, whether they are teenagers or children. And they also understand what is happening to their brain, what is happening to their behavior, what thoughts they have, why these thoughts affect their functioning, how they affect their quality of life.”* (Position 2.161)

She also reports that patients had specifically chosen her as their therapist because they greatly valued the structured and clearly defined procedures of TF-CBT and, in particular, after having already tried other therapeutic approaches, patients had intentionally chosen this treatment method (Position 2.110).

Therapists reported experiencing various emotional and physical reactions during or after the implementation of TF-CBT. These included exhaustion, anger and aggression, as well as intense bodily sensations such as nausea. At the same time, they also described positive emotions, such as joy at being able to help patients process their experiences, and happiness when patients left a session or completed the therapy showing positive changes. To cope with the wide range of often intense emotions, the therapists report using various self-care strategies. Despite their heavy workloads, they state that they do not work on Sundays (even though Sunday is considered a regular workday in some of the countries where the therapists currently reside). In addition, they emphasize the importance of getting sufficient sleep, engaging in physical activity, and ensuring that coffee and food are always readily available. They have also developed highly individualized self-care routines, such as listening to classical music while cooking or cleaning to unwind or engaging in hobbies such as puzzling or sewing. Moreover, exchange with colleagues, such as through supervision within the TF-CBT Ukraine project, was described as beneficial for their own well-being. Participant ID 3.2 describes using stabilization techniques such as breathing exercises or the visualization of a safe place and underscores their importance:

*“When we want to take care of our health, we make appointments with doctors. It's the same with self-care. There must be a sort of self-care schedule (Position 2.370)”.*

Participant ID 3.1 reports similar experiences and adds that her knowledge of Cognitive Behavioral Therapy (CBT) skills has a positive effect on her overall well-being:

*“I can even see how my work, my work in CBT, significantly affects the quality of my life. I mean, you use the same techniques for yourself, and you have a completely different attitude to thoughts, and it's much less exhausting, much easier when you have a good connection with them. So that's also how I deal with it. (Position 3.295)”.*

Nonetheless, participant ID 2.3 also notes that under current circumstances, self-care is particularly challenging. She states:

“*In terms of self-care, nowadays there is a little less sunshine, you know. Somehow the weather is not very conducive to overall wellness. But there are stabilization techniques, I agree (Position 2.368)*”.

### Challenges in treatment

Despite the positive feedback regarding the feasibility and effectiveness, participants reported various challenges in implementing TF-CBT with Ukrainian children and adolescents. The challenges mentioned included various difficulties arising from the ongoing war and its effect on both child, parent and therapist.

#### Ongoing war and the lack of a safe environment

The most frequently mentioned challenge in implementing TF-CBT in the Ukrainian context was the ongoing war, which often meant that a safe environment, could not be guaranteed.

“*Another important thing that we need to keep in mind is that the war is ongoing. I mean, we process a traumatic experience and then it recurs. […] They know based on the sound what [missiles] it is. And then they slip back into that reaction, even though we have processed the previous event. The war is not over. Doing exposure in the midst of the war is a new experience (Position 1.130-131).”*

The ongoing war situation is described by ID 3.3 as a highly emotional burden. She recounts the story of a “[…] *girl from Mariupol, they stayed in that bunker and then they found out about the death of their loved ones (Position 3.68).*" This reality confronted therapists with entirely new challenges, which they also discussed during supervision sessions as part of the accompanying training program. For example, participating therapists reported that during air raid sirens or bomb alarms, clients would panic, crouching on the floor or hiding. In light of the continuous shelling of Ukrainian territories, such intense reactions to the ongoing war represent one of the greatest obstacles to clinical improvement, as “*the child’s psych doesn’t have enough time to fully […] recover*” (ID 1.1., Position 1.134). Therapists reported that they were “*dealing with children who are constantly under stress, just like their parents. Well, maybe there are no bombs flying around, but other cues and triggers arise*” (ID 2.2.; Position 2. 175).

Moreover, several therapists emphasized that the therapeutic goals of parents or children often did not focus on the trauma itself, but rather on issues such as the treatment of other mental health difficulties such as enuresis or school-related difficulties. In the context of the ongoing war of aggression against Ukraine, *“traumatization seems to have taken a back seat. […] It has become part and parcel of their everyday life, this war and the fact that people are dying, this whole situation”* (ID 2.3, Position 2.145).

#### Technical difficulties

Another war-related challenge lies in technical difficulties. As ID 1.1 reported one client had to leave *[name of Ukrainian city removed due to the anonymization]* due to the war and moved to a location with very poor internet connection. As a result, it was logistically impossible to continue therapy. Therapists also reported technical issues such as unstable internet connections due to the power outages: “*You couldn’t do full-fledged exposure because the connection could be interrupted any minute*” (ID 1.1, Position 1.136). However, the extent to which this challenge was perceived varied depending on the infrastructure of the respective workplace. For instance, ID 2.1 explained that her workplace was part of the critical infrastructure: “*Therefore, I was lucky to have uninterrupted power supply in my office. Only once, there was one single time when they turned it off. So, I could have my counselling sessions at work, no problem. Yeah? That's a big advantage for me* (Position 2.261).”

#### Working with caregivers

A commonly reported challenge raised by participants in the focus groups was working with caregivers. For example, participants reported that, due to the war, many parents are themselves traumatized or otherwise psychologically burdened. They are often constantly following the news and, because of their own poor mental health, may negatively impact their children's well-being.

“*Challenges also arise when a child is triggered by their parents. I mean that the parents are in such a bad mental state that the child absorbs their emotions after a session and sinks back into a hole. Circumstances like that. And I can't always talk parents into joining the therapy. In the end, it’s up to them. (ID 1.1., Position 1.130)”*

Caregivers were described as being so preoccupied with their own problems and challenges that, on the one hand, children often assumed the role of the supportive figure within the family dynamic. As a result, caregivers frequently failed to recognize that their children were also struggling. ID 2.2. reported:

*“A lot of children helped their own parents. They were stronger during those bombings. Children helped their parents, supported them. And the first thing parents said was, “No, she is fine. She supported me. She said everything would be fine.” Children often initiated the family’s departure from the occupied territories. The parent-child dynamics got skewed. (ID 2.2., Position 2.312-313)”*

On the other hand, despite receiving psychoeducation and dedicated caregiver sessions as part of the TF-CBT protocol, many caregivers did not fulfill their role in the implementation of TF-CBT and did not apply the parenting skills. ID 3.1 explains this by pointing to a lack of awareness of psychotherapy in Ukraine. *“Parents are more used to just taking pills” (Position 3.229)*. This observation was shared by other therapists, who reported that support offers were often not accepted, and that parents in particular were highly skeptical about psychotherapy. Traumatic experiences and the resulting psychological burden were perceived as a current norm in Ukraine, which led to a lack of awareness among parents and children/adolescents that therapy could be helpful in their situation.

#### Challenges due to cultural aspects

Apart from the previously described, potentially cultural challenges in working with Ukrainian parents, no further specific cultural challenges were reported. ID 1.1. stated: “[…] *no differences in cultural factors or any other national factors. I didn't see that in our program. It was all about ‘here's a problem, this child has this problem, and this is what we do’. That was the scheme. […] And it worked. […] There was nothing about certain things working only for people of specific religions or something like that”* (Position 1.243–246).

### Adaptations for the Ukrainian context

Given the multitude of challenges therapists encountered in delivering TF-CBT to Ukrainian children and adolescents, it is not surprising that certain adaptations were made to ensure successful implementation. However, these were not modifications to the TF-CBT manual itself, but rather adjustments or additional techniques made in response to the challenges described above. The TF-CBT manual was largely adhered to. For example ID 3.1. reported*: “it all depends on the case, […] sometimes you stay for several sessions working on one component* (Position 3.160). The only deviations reported by some therapists concerned a more intensive and prolonged focus on psychoeducation, therapeutic relationship and stabilization, as well as an extension of the overall number of sessions, since in some cases it took longer for clients to feel ready to share their traumatic experiences. In addition, therapy had to be slightly adapted for each individual client. As ID 2.3 reported, for example, breathing techniques did not help all clients, nor did muscle relaxation exercises. It was a “*kind of search within the proposed algorithm*” (Position 2.239) to find the appropriate methods and techniques for each client. “*Some things work well with children, while others don't”* (Position 2.240).

Culturally-based adaptations were reported only by ID 1.1: *“I switch to Russian only if a client is from the Luhansk or Donetsk region, where Russian is their everyday language. As you might know, we do therapy in the language the person thinks in*” (Position 1.14).

#### Working with caregivers

Despite the frequently discussed challenges in working with caregivers, none of the participants reported specific adaptations in how collaboration with caregivers was conducted. In cases where caregivers exhibited noticeable psychological distress, they were, at best, referred to psychologists themselves.

#### Ongoing war and the lack of a safe environment

Due to the ongoing war situation, certain adaptations were necessary to provide both therapists and clients with a greater sense of safety during therapy sessions. For instance, ID 3.2 reported that in the initial sessions, she supplemented the TF-CBT manual by also inquiring about war-related fears, such as fear of explosions or sirens. If significant fear was present, the focus of her therapy would shift toward stabilization, calming, relaxation, and the question: “*How likely is it that you will be okay here?*” In doing so, she aimed to “*give them the opportunity to feel relaxed; only then would I address the traumatic effect, the traumatic event*” (Transcript 3 (Engl): 208).

Since a safe place could not be assumed during the ongoing war situation, therapists reported various adaptations and alternative methods in place of the TF-CBT “safe place” technique. These ranged from imagination exercises exploring what the client’s safe place would look like and which objects and people would be present, to questions such as “*How do you want us to win?*” (Position 2.191), and even “*What happens next? What are we going to do when we win?”* (Position 2.311).

#### Technical difficulties

One therapist, ID 1.1, reported specifically how she proactively addressed the emotional uncertainty caused by power outages during therapy sessions:

*“At the beginning of each session I discuss it with both adults and children, ‘If the Internet goes down, we call back as soon as the power and the Internet come back on, no matter what. Or we text each other if the session is over timewise.’ Because there were situations when I had Internet outages even though I was in another country. […] It reduces their anxiety as they might be thinking, ‘Maybe I've been abandoned’. I had one client like that. He told me, ‘I thought you disconnected halfway through my story because you couldn't take it.’ […] now I always discuss it in advance, ‘If I disappear, it’s not because you told me something wrong […]’ Sort of power supply psychoeducation. (Position 1.136-140)*

## Feedback training program

Participants reported being generally very satisfied with the training program “TF-CBT Ukraine” [24, 25]. One participant stated:

*“The training itself was highly professional. This is something that we lack in Ukraine. Now I have a much better understanding of how to work with children and adolescents with trauma.* (ID 2.1, Position 2.111)“.

They said that being part of the training program made them feel more confident, especially when working with children. Participating therapists particularly appreciated the clear structure of the training, the communication between organizers, trainers, and participants, and regarded every component of the training program as supportive for successfully learning the therapeutic method. As one participant put it:

*“I spend a lot of time learning, but this is probably my first project where there is so much support and guidance. Yes, there are letters. I call them happiness [that’s what I call them]. You know, every time you get one, it's some kind of news that you're waiting for, a reminder that you have a supervision in two days, or a reminder that they are planning to develop roadmaps, and they want to involve you in the process (ID 2.1. Position 2.109)”.*

Participant ID 3.2 emphasized the importance of personal contact as follows:

*“So, actually, the project is valuable not only because it provides knowledge, but also because it provides personal support to us (Position 3.52)”.*

While participants noted that the training involved a high volume of theoretical content at the beginning, they reported becoming increasingly familiar with both the content and the structure over time. Several participants stated that the early and transparent communication of schedules was particularly helpful, as it allowed them to plan accordingly.

Interviewee 2.1 reports that without simultaneous interpretation she would not have been able to participate in the program, as her English proficiency was insufficient. As soon as participation in Ukrainian became possible, she registered immediately (Position 2.50).

The only critical remark regarding the overall training program came from ID 1.1, who commented on the online format:

*“I'm just used to this format. Of course, it would be great if it were in person, if we lived in peacetime, if we had a full day of training with coffee breaks and informal discussions.” (Position 1.63-66).*

However, she went on to note: “*This format works pretty well for me.” (Position 1.67).* Nevertheless, especially at the beginning of the training program, there was a large amount of theoretical input, according to ID 1.1 (Position 1.71). The participating therapists found it very difficult to name a highlight of the training program, since “*I really like the way the program is structured and divided in general, and without any part of the program it would be much more difficult*.” (ID 3.2, Position 3.108). Later she adds: *“It's hard to say, because the whole program was the icing on the cake for me, to be honest. To single it out right away. Well, it's hard for me to say offhand what the highlight was.”* (Position 3.275). However, the supervisions, the voluntary additional workshops, and the provided materials were considered particularly helpful for the learning process. Regarding the supervisions, ID 3.2 reports:

*“The highlight […] I guess it's still supervisions, perhaps. Because I think I have a lot of experience […] of programs that we went through, were trained in. But there were no such long-term year-long supervisions, where you knew that you were allowed to practice clinically and would be supported in this way. […] And you are provided with such support. There used to be intervision groups, there were singular supervisions, but the fact that you are guaranteed to get a year's worth of time and you take cases and you know for sure that you will receive support, I think this is the biggest highlight of this program.”* (Position 3.275–277)

Another key aspect was the additional optional workshops introduced during the course of the project. These addressed supplementary topics relevant to the treatment of traumatized Ukrainian children and adolescents, which were not part of the core TF-CBT training curriculum. As ID 3.2 describes:

*“Theoretically, it is possible to do without those additional seminars that were announced as additional seminars. But this is such a helpful part. I am very happy that it was there too.” (Position 3.108)*

For example, one optional workshop was on the topic on traumatic grief. ID 3.1 remembered:

*“There was a seminar on grief that was very helpful to me. […] topic is an integral part of working with trauma during the war. […]. That is, it became understandable how to clearly recognize this complicated grieving, yes, so the trainers gave very good answers.” (Position 3.88)*

These optional workshops were offered to all project participants, but they were also recorded and made available afterwards, enabling asynchronous learning as well. ID 1.1 reports:

*“and the recordings that I am very grateful for. Because you can’t always listen with concentration, or you can be distracted by something else […], so I listen again. I'm very grateful for that.”* (Position 1.54)

In addition to the individual components of the training program, the materials provided were also considered very helpful:

*“But we are so well provided with methodological material […], everything is uploaded, from workbooks to materials needed for psychoeducation. At each stage, we have a set of materials that we can use. they are divided into folders. Programs that are recommended to us, like a mobile application, in order to implement certain stages of the protocol. It is very convenient in this context.”* (ID 3.X, Position 3.82)

The TF-CBT-specific workbooks in particular were especially helpful in following the therapy protocol (Position 3.83).

### Suggestions for improvement

In order to better tailor training programs to the needs of Ukrainian therapists, suggestions for improvement were collected. Most notably, these included requests for more frequent supervision sessions (twice per month) and more regular additional workshops (at least once a month). ID 2.X explains it as follows: “*The more knowledge you have, the better you understand, the fewer mistakes you make then, right? Well, this is my thing. The more knowledge, the better for me* (Position 2.99).” Specifically, there was a desire for optional workshops on the topics of “sexual abuse against children,” particularly in times of war, and “complex trauma”.

## Discussion

This study presents, for the first time, qualitative data from therapists on the implementation of TF-CBT in an ongoing war context. Overall, despite considerable challenges, the implementation of TF-CBT in Ukraine appears feasible based on therapists’ accounts in the present study. These qualitative findings are consistent with the quantitative outcomes reported by Pfeiffer et al. [25], which demonstrated significant improvements in patient symptoms following treatment. The high demand for trauma-focused interventions for Ukrainian children and adolescents previously reported in earlier studies [26] was once again confirmed by the ease with which participating therapists were able to recruit patients. Particularly noteworthy is that a high degree of treatment fidelity was reported even under highly stressful circumstances affecting not only the children and their parents but also the therapists themselves [10, 31]. While it is important to note that the participating therapists were highly committed to the project and received structured support in the implementation, they nevertheless worked under the extremely challenging conditions of an ongoing war. Thus, this finding not only suggests the cross-cultural applicability of TF-CBT, as previously demonstrated in Thielemann et al. [32], but also adds qualitative evidence to the quantitative shown evidence of the feasibility of TF-CBT in a context of an ongoing war [25]. Therefore, the findings highlight the considerable potential for the use of TF-CBT in crisis and conflict regions worldwide. Similar to the findings reported by Murray et al. [23] in the contextual description, therapists implemented the core elements of TF-CBT. However, certain adaptations were made to implementation techniques in response to the war situation. Despite the absence of a safe environment, previously considered a prerequisite for conducting PTSD-Treatment [30], our findings indicate that TF-CBT can be successfully applied even in situations of ongoing war.

Despite the generally positive findings regarding implementation of TF-CBT during an ongoing war, it is essential that existing training and therapy programs incorporate contextual implementation adjustments that address the specific conditions of an ongoing war in order to ensure long-term implementation. The present study clearly highlights the challenges therapists face when providing trauma-focused therapies during a persistent threat situation. Both, protocol-consistent flexibility on the part of therapists, openness on the part of patients and their caregivers, and contextual implementation adjustments by trainers and supervisors are required to ensure alignment between specific needs and available services. In the present project, for example, the introduction of simultaneous translation proved to be a particularly successful contextual implementation adjustment. It might not only substantially increased the utilization of the program but might also have improved the overall quality of the training. By providing translation, language barriers were reduced, which significantly enhanced communication between trainers and supervisors and the therapists. This measure likely contributed to the high level of treatment fidelity reported [25]. In addition, further contextual implementation adjustments, such as optional workshops addressing topics and the therapists’ needs, allowed the project to respond flexibly to challenges arising during the implementation. Continuous contact between the project team and the participants allowed for timely responses to these needs, which proved to be essential for the overall success of the program. Together, these contextual implementation adjustments, alongside protocol-consistend flexibility in delivering TF-CBT, may enable therapists successfully learn and implement TF-CBT even under particularly challenging circumstances. Therefore, it is essential to respond to the specific local conditions and requirements in order to create training opportunities that are as accessible as possible. As some therapists reported own psychological distress after TF-CBT sessions it seems crucial to offer ongoing case consultation and supervision, thereby also focusing on the mental wellbeing of the therapists by programs such as the PRACTICE skills course [27].

Working with caregivers remains a major challenge. Therapists in this study did not report specific contextual implementation adjustments that had already been implemented to improve the inclusion of caregivers in TF-CBT treatment. Notably, although the absence of caregiver participation might be largely driven by contextual barriers, reduced caregiver involvement may also represent a modification affecting a core treatment element of TF-CBT, highlighting the importance of addressing these barriers in future implementation efforts. This may be attributable to several factors. On the one hand, it is conceivable that parents themselves are heavily burdened by structural barriers, not least the war situation, not only by own psychosocial burden [17] but also by the general wartime instability, own psychological burden and related factors such as displacement-related stress. On the other hand, broader societal and cultural factors, including limited mental health literacy and stigma toward mental illness, may further influence caregiver engagement in treatment [28]. These findings are consistent with broader evidence from conflict-affected settings indicating that mental health literacy and stigma significantly shape attitudes toward psychological disorders and help-seeking behavior [1]. Recent studies from Ukraine similarly highlight persistent mental health stigma and evolving patterns of help-seeking during the ongoing war [11, 21]. In addition, research on parenting in contexts of armed conflict suggests that exposure to war-related stressors may influence caregiving practices, emotional availability, and interpretations of child psychological distress [2, 9].

It is therefore highly necessary to increase societal awareness of the relevance of mental disorders and of the effectiveness of evidence-based therapeutic methods, alongside or at least as an alternative to psychopharmacological treatment. Psychoeducational campaigns (e.g. [12]) and other measures aimed at reducing stigma toward mental illness (e.g. [8]) should therefore be increasingly implemented in order to improve collaboration between therapists and the family system.

## Limitations

The findings of the present study should be interpreted in light of several limitations. A substantial sample bias cannot be ruled out. Participants in the study were particularly active therapists involved in the “TF-CBT Ukraine” project, who had distinguished themselves through regular and highly engaged participation in supervision sessions. This sampling strategy was chosen in the hope of achieving the highest possible participation rate in the focus groups in order to facilitate in-depth and meaningful discussions. However, it likely resulted in the overrepresentation of particularly motivated, committed, and well-supported therapists. Consequently, the findings may not be transferable to the broader population of therapists participating in the project, particularly those with lower levels of engagement, different implementation experiences, or greater challenges in applying TF-CBT in routine practice. Nevertheless, in the larger TF-CBT Ukraine project, participating therapists demonstrated similar demographics as the participants in the present subsample, high treatment effectiveness, low dropout rates, high treatment attitudes and positive attitudes toward evidence-based practice [18, 25], there are reasons to assume that the positive experiences reported in the focus groups were not limited to the selected participants but were also reflected in the broader project outcomes. Furthermore, all participating therapists have been shown as particularly motivated and resilient [18].

In addition, the symptom improvements reported in this study are based solely on therapists’ subjective reports. However, these findings are consistent with the objective measures previously demonstrated in the evaluation study of the “TF-CBT Ukraine” project [25]. Furthermore, one participant reported symptom worsening following exposure work. This might be an important consideration for the implementation of TF-CBT in an ongoing war context, however, this aspect was mentioned only briefly and by only one therapists. In addition, one of the focus groups was conducted with only one participant, as the other participants did not show up at the agreed-upon time. As a result, this was more like an interview situation, and there was no opportunity for discussion among colleagues. However, the same interview guide was used for all discussions. Furthermore, other contextual factors may have influenced the implementation of TF-CBT, such as referral pathways, financial resources, or partnerships with other institutions or nongovernmental organizations. Lastly, because focus group participation was anonymous and contextual characteristics were not collected at the individual participant level, the influence of specific organizational settings on implementation experiences could not be examined.

## Implications

The present study clearly demonstrates that TF-CBT can be implemented successfully in an ongoing war context with only minor adaptations, although a structured and intensively supervised training program is required. TF-CBT should therefore be further disseminated in other conflict- and crisis regions. Political stakeholders need to place greater emphasis on the relevance of such measures and provide, among other forms of support, adequate financial resources in order to sustainably improve the mental health of children and adolescents worldwide. At the same time, accompanying research remains necessary, as context-specific adaptations to training programs may be required.

Online formats introduce new challenges but also create many new opportunities to further advance the global dissemination of trauma-focused therapeutic methods. In regions with little evidence-based treatments implemented so far, they might represent an important solution. Therefore, scientific evaluations of digital training concepts are crucial.

Self-care among therapists working in these highly demanding circumstances is of particular importance. This is not only reported by the participating therapists in this study but is also reflected in the broader scientific discussion on secondary traumatization and the overall professional quality of life of therapists [4]. At the beginning of the war of aggression, the Ukrainian therapists in this project demonstrated remarkable resilience [18]. However, this resilience must be continuously maintained and stabilized through ongoing self-care activities. Programs such as a PRACTICE skill course for therapists [27] should therefore be implemented broadly for therapists working in crisis and conflict regions so that they can learn strategies for effective self-care. Only therapists who themselves maintain good psychological well-being possess sufficient mental capacity to support and improve the mental health of traumatized children and adolescents.

Several lessons learned from the implementation process may inform future dissemination efforts in conflict-affected settings. First, delivering the training in the local language proved essential for participant engagement and comprehension, underscoring the importance of translating all training materials. Second, recruitment strategies should take cultural and contextual factors influencing participation into account. In our experience, initial registration did not always reflect a firm intention to complete the training. Clearly communicating expectations regarding full attendance and obtaining explicit commitment from participants prior to the start of the program improved training completion rates. Third, the core training should be complemented by context-specific content, including working with ongoing trauma exposure, digital service delivery, assessment procedures, and foundational competencies in child and adolescent mental health for therapists with limited prior experience in this population. Finally, as therapists themselves are exposed to the consequences of war, implementation programs should incorporate structured self-care components and ensure that therapist well-being is routinely addressed during supervision.

The “TF-CBT Ukraine” project once again illustrates how cooperation between international experts and local partners can lead to the development of sustainable structures within the respective regions. As a result of the project, a follow-up initiative has now emerged directly within Ukraine, in which participants from the original “TF-CBT Ukraine” project, now being internationally certified TF-CBT trainers, are training additional Ukrainian therapists in TF-CBT. However, a participatory approach is needed not only in practical implementation but also in research in order to adapt programs appropriately to the context-specific realities faced by therapists, children, and adolescents in affected regions. Furthermore, it is essential to give a voice to those implementing these methods under challenging conditions through increased qualitative research. This study represents one of the first qualitative investigations focusing on therapists’ perspectives in wartime settings. In the future, children, adolescents, and parents should also be included in qualitative studies so that their perspectives can be more strongly represented in research and experiences related to trauma therapy and war can be better understood.

## Conclusion

This study is encouraging not only in light of the now more than four-year-long war of aggression by Russia against Ukraine, but also clearly demonstrates the possibility of providing evidence-based trauma therapy at an early stage in other conflict and war regions, rather than waiting for the often unpredictable end of a conflict. Such an approach could contribute to reducing the currently very high rates of PTSD in populations living in war and crisis regions [3] at an early stage and, ideally, help prevent complex and chronic trajectories [29].

## Supplementary Information

Additional file 1.

## Data Availability

The anonymized dataset used for this study is available from the corresponding author upon reasonable request. The used semi-structured interview guideline can be found in the supplementary material of this publication.

## Abbreviations

CBT: Cognitive behavioral therapy
TF-CBT: Trauma-focused cognitive behavioral therapy
PTSS: Post-traumatic stress symptoms
PTSD: Post-traumatic stress disorder
