# Supplementary material for: “When the safe place is not safe”: therapists’ perspectives on implementing TF-CBT during the war in Ukraine
Source: Child Adolesc Psychiatry Ment Health. 2026 Aug 16;20:111. doi: 10.1186/s13034-026-01142-0 (PMC13508406; doi:10.1186/s13034-026-01142-0)
Supplement: Supplementary file 1 [file 13034_2026_1142_MOESM1_ESM.pdf]

TF-CBT Ukraine Project: Guide for Focus groups with TF-CBT therapists

- Method: Focus-Groups with 5-7 participants
- Aim:
    - Learn about the therapists' experiences with the TF-CBT treatment in Ukraine
    - Learn about the therapists' experiences with the program
    - Evaluate the program's feasibility from a therapist perspective

Introduction for the researcher

Below you find a table how to structure the TF-CBT Ukraine focus groups/interviews with Ukrainian therapists. The table is structured as follows:

1. In the first column we defined topics to be addressed in the focus groups.
2. The second column comprises the main questions to explore the topics.
3. The third column of the table contains suggestions for underlying themes to flexibly address when you think corresponding content presents itself during your focus group conversations. These can be used to guide more in-depth exploration of participants' answers across underlying dimension. They are not specifically tied to certain questions and can be asked whenever the topic is raised.

Please make sure that there is sufficient time to discuss TOPIC 3, as it is the main focus of this group discussion!

An appendix at the end provides you with suggestions for follow-up and probing questions to use during your focus group discussion in order to facilitate or broaden the discussion.

TF-CBT Ukraine: Focus group guideline with therapists

<table>
<tr><th>Topic</th><th>Questions</th><th>Themes for in-depth exploration</th></tr>
<tr><td colspan="3">Suggestion for an introduction: 'Thank you very much for participating in the TF-CBT Ukraine program and for being in this focus group today. Within the TF-CBT Ukraine program, you learnt principles of TF-CBT and received monthly case consultations to discuss your TF-CBT treatment cases. Additionally, you were invited to attend extra sessions to learn more about trauma assessment, traumatic grief or caregiver involvement, among others topics.
This is the very first program implementing an evidence-based treatment for traumatized children in Ukraine. Therefore, we are very interested in learning from your experiences with the treatment and the overall program.
We are interested in positive as well as challenging experiences you might have encountered during the program.

After the introduction there will be a small round where everyone can introduce themselves.</td></tr>
<tr><td>Topic 1:

overall experience with the program (OPENING QUESTIONS)</td><td>1. What was your general motivation/ reason for participation?

2. What were your expectations when you signed up for the program?

3. How did you learn about the project?

4. Can you tell us about your experiences of participating in TF-CBT Ukraine?</td><td></td></tr>
<tr><td>Topic 2:

TF-CBT Ukraine Program</td><td>1. What do you like about the program?
2. Do you have any suggestions for improvement of the program?

CLOSING QUESTIONS:
1. What was your highlight of the TF-CBT Ukraine project?</td><td>PERSONAL
- Did the program meet your expectations?
- Do you have any further wishes for contents?

ORGANISATORIAL
- Do you feel supported by the program organizers (Elisa, Maike, Vitalii)?
- What do you think could be changed in this program to better meet your needs/expectations?
- What do you like about the
    o Web-training
    o Manual</td></tr>
</table>

		o Basic training<br>o Case consultations<br>Optional sessions
Topic 3:<br><br>Treatment	2. Have you already started treating your own patients with TF-CBT since you signed up for the program? If yes, can you tell us about your experiences?<br><br>3. If yes, when you compare the mental well-being and functioning of the patient before and after the treatment: Did you notice any changes in your patients?<br><br>*4. If there are therapists present who did not treat patients until now:*<br>What were the main challenges/reasons why you were not able to treat patients?	TREATMENT CONDITIONS<br>- How did you manage to **find patients for TF-CBT**?<br>o What were challenges/barriers in the recruitment process?<br>- How were the therapy sessions with the **caregivers** for you?<br>- Which application mode(**digital or in person**?) did you use for treating patients<br>o What was more feasible for you?<br>o What way do you think might be easier/better for patients?<br><br>KNOWLEGDE/FEELINGS<br>- How do you feel during the TF-CBT Treatment?<br>o Did you have sufficient **knowledge/materials** to implement the manual?<br>o How helped your **previous qualification**/ training (outside of the program) during the learning and implementation process? Are your **previous experiences** in psychotherapy in line/stand in contrast with the TF-CBT treatment?<br><br>TF-CBT FEASABILITY<br>- Do you think that the TF-CBT treatment works well in the Ukrainian context?<br>- What do you think can be changed in the way TF-CBT is structured, to better accommodate the needs of your patients?<br>- Did you make any **adaptations** to the (current) context<br>o Due to the ongoing war? If yes, which ones?<br>o Due to cultural/ country-specific factors? If yes, which ones?<br>-

Appendix – suggestions for questions to facilitate/broaden your FG discussion

A. Helpful questions for the facilitation

- *"Please tell me more"*
- *"Please give me an example"*
- *"Please help me understand"*
- *"What do you think caused the problem?"*
- *"How did you feel in the situation?" (for example, during the treatment, the basic training, the case consultation).*
